# Convulsions in children hospitalized for acute gastroenteritis

**DOI:** 10.1038/s41598-021-95202-4

**Published:** 2021-08-05

**Authors:** Moti Iflah, Eias Kassem, Uri Rubinstein, Sophy Goren, Moshe Ephros, Dani Cohen, Khitam Muhsen

**Affiliations:** 1 School of Medicine, Sackler Faculty of Medicine, Tel Aviv University, Tel Aviv, Israel; 2 Department of Pediatrics, Hillel Yaffe Medical Center, Hadera, Israel; 3 Department of Pediatrics, Laniado Medical Center, Netanya, Israel; 4 Department of Epidemiology and Preventive Medicine, School of Public Health, Sackler Faculty of Medicine, Tel Aviv University, Ramat Aviv, 6139001 Tel Aviv, Israel; 5 Department of Pediatrics, Carmel Medical Center, Haifa, Israel; 6 Faculty of Medicine, Technion-Israel Institute of Technology, Haifa, Israel

**Keywords:** Epidemiology, Paediatric research, Medical research

## Abstract

The study aim was to examine possible correlates of convulsions in children hospitalized for acute gastroenteritis (AGE). Data collected in a prospective study of AGE hospitalizations in children aged 0–59 months in 3 hospitals in Israel during 2008–2015 were analyzed. Stool samples were tested for rotavirus using immunochromatography and stool culture was performed for the detection of *Salmonella*, *Shigella* and *Campylobacter* We compared clinical and demographic characteristics of children hospitalized for AGE who had convulsions (n = 68, cases) with children hospitalized for AGE without convulsions (n = 3505, controls). Age differed between children with and without convulsions (*p* = 0.005); the former were mostly toddlers aged 12–23 months (51%) compared to 30% of the control group. A higher percentage of cases tested positive for *Shigella* (11% vs. 4%, *p* = 0.002), the opposite was found for rotavirus (2% vs. 30% *p* < 0.001). A multivariable model showed that body temperature (OR 2.91 [95% CI 1.78–4.76], *p* < 0.001) and high blood glucose level (> 120 mg/dL) (OR 5.71 [95% CI 1.27–25.58] *p* = 0.023) were positively related to convulsions in children with AGE, while severe AGE (Vesikari score ≥ 11) was inversely related with convulsions (OR 0.09 [95% CI 0.03–0.24], *p* < 0.001). *Conclusion:* Elevated body temperature is associated with convulsions in children with AGE, but not severity of AGE, while hyperglycemia might reflect a neuroendocrine stress reaction to convulsions, AGE or both.

## Introduction

Acute gastroenteritis (AGE) is a leading cause of morbidity in young children globally, and it is a leading cause of child mortality in low and middle-income countries^1^.

The clinical manifestations of AGE include abdominal pain, vomiting, diarrhea and fever^2^. Some patients with AGE also develop convulsions^3–11^ that might be febrile or afebrile convulsions. Some authors have defined the term “benign convulsions” in children with mild gastroenteritis that occur in previously healthy children in the absence of fever and dehydration^12–14^. Specific enteric pathogens have been linked to convulsions in children, such as rotavirus, norovirus^4,5^, *Campylobacter* and *Shigella* ^8,10^. Vaccines against rotavirus have been available since 2006 and were introduced into national immunization programs in more than 100 countries^15^. In Israel, universal rotavirus immunization was introduced to the national immunization program in December 2010 using the pentavalent rotavirus vaccine^16^. The introduction of universal rotavirus immunization was followed by significant and consistent reduction in the burden of all-cause AGE and rotavirus gastroenteritis^16–21^. Currently, there are no licensed vaccines for other enteric pathogens.

Convulsions in children with AGE were studied previously^4,6,7,10,11,22–29^, but given the variation in the study design and patient population across previous studies, convulsions in children with AGE remain not fully characterized. For example, the lack of a comparison group of children without convulsions^6,22–29^ hindered the understanding of possible correlates of convulsions in AGE patients. Moreover, most studies focused on viral AGE^4,11,23,24^, or pre-selected children with normal electrolyte balance, or with afebrile convulsions^22,29^. Accordingly, the aim of the current study was to compare demographic, clinical and laboratory findings of children with AGE who had convulsions during their illness with those who did not. Our main hypothesis was that bacterial enteropathogens are more common among children with AGE and convulsions than in those who do not develop convulsions. In addition, aberrations in levels of blood electrolytes might be more prevalent in those with convulsions.

## Materials and methods

A case–control study was undertaken using data collected in the framework of a prospective surveillance network of hospitalizations for AGE among children aged 0–59 months in 3 hospitals in Israel: Carmel in Haifa, Hillel Yaffe in Hadera and Laniado in Netanya, spanning 2008–2015. Details on the study have been described elsewhere^17,19–21,30^. Briefly, the sampling frame included children aged 0–59 months hospitalized with acute diarrhea (< 7 days). Inclusion criteria were 3 or more watery stools during a 24-h period and parental consent to take part in the study. Children with prolonged diarrhea (> 7 days) before admission were excluded from the study. Data were collected via parental interviews using a structured questionnaire and from medical records, following the World Health Organization generic protocol^31^. Data were collected on demographics: age in months (analyzed as a categorical variable 0–11 months, 12–23 months and 24–59 months), sex, population group and residential socioeconomic status rank. Socioeconomic status (SES) was classified based on the socioeconomic rank of place of residence at the level of the community (town/city/village), as determined by the Central Bureau of Statistics. The ranks are on a scale from 1 to 10, with higher ranks representing a higher SES. This is an aggregative SES index calculated using multiple demographic, social and economic factors, including financial resources of the residents, housing conditions, density, motorization level, educational level and employment profile^32^. This variable was categorized by tertiles as following: ranks 1–3 were classified as low SES, ranks 4–6 as intermediate SES and ranks 7–10 as high SES. In addition, we obtained data on clinical signs and symptoms, including fever (measured body temperature greater than 38 °C) and convulsions on admission.

The definition of convulsions was based on documentation in medical records. In the current study, clinical and demographic characteristics of all children hospitalized for AGE with convulsions (n = 68, cases) and children hospitalized for AGE without convulsions (n = 3505, controls) were compared.

Detailed data were collected on a sub-sample of children who were hospitalized at Hillel Yaffe hospital; this included all 47 cases and 114 randomly selected controls. The controls were selected among hospitalized children with AGE who did not have convulsions. Group matching was performed by age and calendar year of admission in this sub-study, in which the controls were selected from strata of age (0–11, 12–23 and 24–59 months) and year of admission (2008–2015). These children were analyzed for additional data from medical records on birth weight, gestational age at birth, on laboratory test results on admission (complete blood count, plasma glucose, sodium, potassium and C-reactive protein [CRP] levels), and characteristics of the convulsions. For some patients, blood samples were not obtained in fasting condition. Only 3 children had a glucose level > 150 mg/dL, therefore for the classification of high blood glucose levels we used the cutoff value of > 120 mg/dL that corresponded to the 90th percentile. Severity of AGE was assessed using a modified Vesikari score^33^, based on clinical symptoms (number of diarrhea days, number of stools, number of vomiting days, number of vomiting episodes, fever and treatment – hospitalization). Scores of ≥ 11 were considered as severe AGE and scores < 11 were considered as non-severe AGE.

The available sample size of 68 cases and 3505 controls yielded a statistical power of 87% to identify significant differences between cases and controls in the presence of bacterial pathogens in stool (16% in the control group, odds ratio [OR] of 2.5).

### Laboratory methods

A stool sample was collected from the participants within the first 48 h of admission. Stool specimens were tested for rotavirus antigen by immunochromatography (Rotavirus Dipsticks, Hylabs Rehovot and Novamed, Jerusalem, Israel) according to manufacturers’ instructions. The presence of *Salmonella*, *Shigella* and *Campylobacter* in stool was tested by culture using standard microbiology tests.

### Statistical methods

The sample was described using minimum–maximum values, median and interquartile range for variables with skewed distribution, and frequencies and percentages for categorical variables. A bivariate analysis was performed using the Mann Whitney *U* test for variables with skewed distribution, and the chi square test and Fisher exact test as appropriate for the categorical variables. Logistic regression models were fitted to assess correlates of convulsions. Odds ratios and 95% confidence intervals (CI) were calculated for each independent variable. *P* < 0.05 was considered statistically significant. For variables with missing values (Supplementary Table 1), multiple imputation was performed^34,35^. Sensitivity analyses were performed using the complete case analysis strategy and by limiting the analysis to children with fever (body temperature > 38 °C). Data were analyzed using SPSS version 25 (IBM, New York, United States).

### Ethics approval

The study protocol was approved by Institutional Review Boards of the participating hospitals and the Ministry of Health. Parents signed a written informed consent form. The study was conducted in accordance with the Declaration of Helsinki, all procedures were performed in accordance with local guidelines and regulations. The protocol of the current study was approved by the Ethics (Helsinki) Committee of Hillel Yaffe Medical Center (Project identification 0133-19-HYMC).

## Results

Overall, 3573 children (1923 [54%] males) hospitalized for AGE were included in the study. The median age was 12.0 months (interquartile range 16.0). Overall, 68 (1.9%) children had AGE with convulsions. There was a significant difference in age between children with and without convulsions (*p* < 0.001). The percentage of infants aged 0–11 months was lower among children with convulsions compared to those without convulsions (19% vs. 47%). The respective percentages of toddlers aged 12–23 months were (51% and 30%), while the respective percentage of children aged 24–59 months was 30% and 23%. No significant differences were found between children with and without convulsions regarding sex, population group or SES (Table 1).

**Table 1:** Socio-demographic characteristics of children hospitalized for acute gastroenteritis with and without convulsions.

	Cases (n = 68)	Controls (n = 3505)	*P* value^a^
Median age (IQR), months ^b^	17.5 (15.5)	12.0 (16.0)	< 0.001
Age, months			< 0.001
0–11	13 (19%)	1647 (47%)	
12–23	35 (51%)	1059 (30%)	
24–59	20 (30%)	799 (23%)	
Sex			0.168
Males	31 (46%)	1892 (54%)	
Females	37 (54%)	1612 (46%)	
Population group			0.331
Arabs	23 (34%)	1389 (40%)	
Jews	45 (66%)	2115 (60%)	
SES of place of residence ^c^			0.074
1–3 (Low)	14 (23%)	1150 (36%)	
4–5 (Intermediate)	38 (62%)	1563 (48%)	
6–10 (High)	9 (15%)	527 (16%)	

^a^*P* value was obtained by the chi square test, unless specified otherwise.

^b^IQR: interquartile range; *P* value was obtained by the Mann–Whitney *U* test.

^c^SES: Socioeconomic status. Information on residential SES was missing for 7 (11%) cases and 264 (8%) controls and on sex and population group for 1 control.

Nearly one-third (29%) of the cases tested positive for any bacterial enteropathogen compared to 16% of controls (*p* = 0.008). *Shigella* spp. was detected in 11% of cases vs. 4% of controls (*p* = 0.002). There was no significant difference between the groups in positivity for *Campylobacter* or *Salmonella* spp*.* Rotavirus was detected less frequently among the cases (2%) compared to 30% of controls (*p* < 0.001) (Table 2). This inverse association was consistent during the years before and after the introduction of universal rotavirus vaccination in Israel (*p* = 0.2 by Breslow = Day test for homogeneity).

**Table 2:** Microbiologic results of children hospitalized for acute gastroenteritis with convulsions (cases) and without convulsions (controls).

	Cases (n = 63)	Controls (n = 2972)	*P* value ^a^	OR (95% CI)^b^
Bacterial enteropathogen^c^	18 (29%)	479 (16%)	0.008	2.08 (1.19–3.63)
*Shigella*	7 (11%)	106 (4%)	0.002	3.38 (1.50–7.59)
*Campylobacter*	10 (16%)	286 (10%)	0.098	1.77 (0.89–3.52)
*Salmonella*	2 (3%)	91 (3%)	0.719	1.04 (0.25–4.31)
Rotavirus (overall)^d^	1/45 (2%)	665/2311 (29%)	< 0.001	0.05 (0.01–3.76)
Pre-universal rotavirus vaccination (2008–2010)	1/12 (8%)	433/1102 (39%)	< 0.001	
Universal rotavirus vaccination (2011–2015)	0/33 (0%)	232/1209 (19%)	< 0.001	

^a^*P* value was obtained by the chi square or Fisher's exact test.

^b^Odds ratio (OR) and 95% confidence intervals (CI) were obtained using bivariate logistic regression models, comparing children with the pathogen to those without the pathogen.

^c^The detection of *Shigella*, *Salmonella* or *Campylobacter* spp. in stool culture.

^d^Rotavirus was tested in 45 cases and 2311 controls.

### Sub-sample with detailed clinical information and tests results

A medical chart review was performed for all 47 children with AGE and convulsions and the 114 controls with comparable demographic profiles (Supplementary Table 2). Two of the case children had a diagnosis of epilepsy before the current admission and were treated regularly with Valproic acid (Depalept). The rest of the children had no neurological diseases. None of the patients had a diagnosis of meningitis or encephalitis.

Most convulsions were described by the treating physician as generalized tonic clonic seizures lasting between 1 and 20 min. In some children, convulsions were followed by a postictal period. Lumbar puncture was performed on admission in 6 patients; CSF culture and enterovirus tests were negative in all. Electroencephalography (EEG) was performed in 12 patients and in 11 it was interpreted as normal. The diagnosis code of febrile convulsions appeared as the only diagnosis code in 16/47 (34.0%) of the patients, in 19/47 (40.4%) of the patients the diagnosis codes were AGE and convulsions, 9/47 (19.1%) had a diagnosis code of viral infection (with or without convulsions), while 3/47 (6.4%) had other diagnosis codes. Among 31/42 patients (74%) the convulsions were described by the physician as simple febrile convulsion, and in 11/42 (26%) as complex convulsions.

The median gestational age at birth was slightly lower among cases than controls (39.0 vs. 39.8 weeks, *p* = 0.045), but there was no significant difference between the groups in the percentage of premature born children (< 37 weeks), 17% and 10%, respectively (*p* = 0.140). No significant difference was found between the groups in birth weight (median 3109 and 3192 g, respectively, *p* = 0.254).

Overall 96% of the cases and 73% of the controls had fever (> 38 °C), *p* = 0.001, and the median body temperature was higher among cases than controls. The median Vesikari score was significantly lower among cases than controls: 9 vs. 12, *p* < 0.001. On admission, children with convulsions had a higher median white blood count of 15.5 × 10^3^ per µL (range 5.4–31.4 × 10^3^ per µL) than controls 12.2 × 10^3^ per µL (range 4.9–35.0 × 10^3^ per µL), (*p* = 0.019). The median plasma glucose level was higher (*p* < 0.001) among children with convulsions (103.4 mg/dL [range 79–186]) than the controls (87.4 mg/dL [range 30–139]), (Table 3). High blood glucose levels, defined as glucose > 120 mg/dL, was more common among cases than controls 23% vs. 8%, *p* = 0.013). Serum sodium was lower among children with convulsions compared to those without (median 136.9 mEq/L [range 129.2–145.0]) vs. 138.0 mEq/L, [range 128.7–144.0]) (*p* = 0.002). Hyponatremia (serum sodium level < 135 mEq/L) on admission was found among 26% and 14% of the cases and controls, respectively, but the difference was not statistically significant (*p* = 0.059). No significant differences were found between the groups in hemoglobin, platelets, potassium or CRP levels (Table 3).

**Table 3:** Clinical characteristics of children hospitalized for acute gastroenteritis with convulsions (cases) and without convulsions (controls).

	Case (n = 47)			Controls (n = 114)			*P* value ^a^
	Min	Max	Median (IQR)	Min	Max	Median (IQR)	
Body temperature, °C	36.2	42.0	40.0 (1.0)	36.0	42.0	39.0 (2.0)	< 0.001
Gestational age at birth, weeks	31.0	41.0	39.0 (2.9)	35.0	42.0	39.8 (2.5)	0.045
Birth weight, grams	1260	4600	3109 (956)	1750	4600	3192 (791)	0.254
Vesikari score	6	15	9 (4)	5	17	12 (4)	< 0.001
White blood count × 10^3^ per µL	5.4	31.4	15.5 (11.7)	4.9	35.0	12.2 (7.4)	0.019
Hemoglobin, mg/dL	9.2	15.2	11.7 (1.0)	8.8	16.0	11.8 (1.6)	0.226
Platelets × 10^3^ per µL	192	552	317 (119)	31	757	355 (124)	0.089
Glucose, mg/dL	79.0	186.0	103.4 (23.4)	30.0	139.0	87.4 (25.6)	< 0.001
Potassium (K), mEq/L	3.3	5.8	4.3 (0.7)	3.3	6.9	4.4 (0.7)	0.647
Sodium (Na), mEq/L	129.2	145.0	136.9 (3.4)	128.7	144.0	138.0 (4.0)	0.002
C reactive protein,mg/L	0.4	116.3	17.0 (31.5)	0.2	310.6	9.3 (31.4)	0.087

^a^*P* value was obtained by the Mann–Whitney *U* test given skewed distributions for most variables in the table.

*IQR* interquartile range, *Min* minimum value, *Max* maximum value.

A multivariable logistic regression model was conducted including the variables hyponatremia, high blood glucose level (glucose > 120 mg/dL), CRP levels, severity of AGE (Vesikari score ≥ 11), highest body temperature and gestational age at birth as the independent variables. This model showed positive associations between body temperature and convulsions (adjusted OR 2.91 [95% CI 1.78–4.76], *p* < 0.001) for each one degree Celsius increase in measured body temperature. A positive association was found between high blood glucose level and convulsions (adjusted OR 5.71 [95% CI 1.27–25.58], *p* = 0.023). Having a severe AGE (Vesikari score ≥ 11) was inversely related with convulsions (adjusted OR 0.09 [95% CI 0.03–0.24], *p* < 0.001). The association between hyponatremia and convulsions was not statistically significant (adjusted OR 3.10 [95% CI 0.93–10.36], *p* = 0.066), as well as the associations of CRP and gestational age at birth with convulsions (*p* = 0.166 and 0.217, respectively) (Table 4). The complete case-analysis showed comparable results (Supplementary table 3) as well as when limiting the analysis to children with fever (body temperature > 38 °C) (Supplementary table 4).

**Table 4:** Multivariable logistic regression model of the adjusted associations of clinical characteristics with convulsions in children hospitalized for acute gastroenteritis.

Variable	Adjusted OR (95% CI)	*P* value
Gestational age at birth, weeks	0.80 (0.62–1.04)	0.088
C-reactive protein, mg/L	0.99 (0.98–1.00)	0.114
Body temperature, °C	2.91 (1.78–4.76)	< 0.001
Hyponatremia (Sodium < 135 mEq/L)	3.10 (0.93–10.36)	0.066
High plasma glucose level (glucose > 120 mg/dL)	5.71 (1.27–25.58)	0.023
Severe gastroenteritis (Vesikari score ≥ 11)	0.09 (0.03–0.24)	< 0.001

*CI* confidence intervals, *OR* confidence intervals.

The duration of hospitalization ranged between 1 and 7 days (median = 2) in children with AGE and convulsions and between 1 and 13 days (median = 2) in the controls (*p* = 0.6, by the Mann Whitney *U* test). All patients were discharged to home.

## Discussion

In this study, nearly 2% of children hospitalized for AGE developed convulsions. Convulsions with AGE differed significantly according to age, with children aged 12–23 months comprising nearly half of the cases. Children with convulsions and AGE also had a higher percentage of bacterial AGE, mainly shigellosis than the control group. This finding is consistent with other reports of seizures in children with shigellosis^8,36–38^. A case–control study from Iran compared between children hospitalized for AGE with and without convulsions, and showed a higher frequency of *Shigella* spp. in stool culture of children with convulsion than those who did not have convulsions^7^. Convulsions might develop in all *Shigella* spp.^39^. In *Shigella dysenteriae* Shiga toxin (Stx) is a main neuro-virulent factor. Animal studies have demonstrated that Stx might facilitate seizures via mechanisms involving tumor necrosis factor alpha (TNF alpha), interleukin l beta and nitric oxide^40–42^, and it might act together with lipopolysaccharide (LPS) in the induction of neurologic disorders^43^ that altogether interact with host response^44^. In Israel, *Shigella sonnei* is the most common serotype^45^. Recent evidence has demonstrated that *S. sonnei* may produce Shiga toxin 1 and 2^46–48^. Thus, the increased prevalence of *Shigella* in AGE patients with convulsions vs. those without convulsions might be due to possible neurovirulent qualities of *Shigella* LPS and maybe other toxins.

Limited evidence suggests that *Campylobacter* might play a role in convulsions among patients with AGE^10,28^; however, this association was not statistically significant in our study, possibly due to the small sample size. Convulsions in our study were less common in children who tested positive for rotavirus vs. those who tested negative. This was somewhat surprising given previous reports suggesting that rotavirus might be involved in the etiology of convulsions in children with AGE^22–24,29,49^. Other infectious agent such as enteroviruses and Parechoviruses might cause convulsions as part of infections involving the central nervous system, namely meningitis or encephalitis, however none of the patients in our study had meningitis or encephalitis. Measured body temperature was positively related to convulsions in our patient sample, suggesting that fever might be the trigger of convulsions. Interestingly, having a severe AGE as defined by a Vesikari score of ≥ 11 was inversely related to convulsions in our study. Vesikari score measures aspects of dehydrating diarrhea, thus suggesting that the mechanism of developing convulsions during a diarrheal episode is less likely explained by dehydration.

Mostly previous studies on convulsions among children with AGE focused on patients with mild diseases, without fever or dehydration or electrolyte imbalance^4,6,22^ or those with viral gastroenteritis (e.g. rotavirus or norovirus)^5,11,23,26,27,29^, while we did not apply such selection criteria to better understand the occurrence of convulsions among children with AGE. Accordingly, direct comparison between our and others' findings might be limited. A study from Iran^7^ that employed a similar design to our study, showed higher mean body temperature and less severe dehydration among children with AGE and convulsions compared to children who had only AGE without convulsions. A multicenter study conducted in Japan compared between children with viral AGE who had febrile convulsions and those with afebrile convulsions^22^, showed significantly lower serum sodium level in those with febrile vs. afebrile convulsions. In our study, the positive association between hyponatremia and convulsion was borderline statistically significant.

A study that was conducted in Bangladesh among patients with shigllosis showed that a shorter duration of diarrhea, higher body temperature, higher median weight-for-age, increased proportion of immature leukocytes, higher serum potassium, and lower serum sodium levels were independently associated with the development of convulsions in patients < 15 years old^50^.

We found a significant positive association between high plasma glucose and convulsions in children with AGE. High plasma glucose might be a part of a neuroendocrine response to convulsions, AGE or both. Hyperglycemia in children with acute illness including diarrhea has been described and was attributed to stress^51–54^. Few case reports and case series have reported stress hyperglycemia in children with febrile convulsions^54,55^. Valerio et al. showed that stress hyperglycemia was more common in children with febrile convulsions than children with fever alone or with other diagnoses^56^. Thus, the higher glucose levels in the case group might represent a greater stress response. Others have shown that stress hyperglycemia might be a risk factor for recurrence of febrile convulsions^57^. Since blood test were obtained on admission. It is highly likely that blood tests on admission do not represent pre-convulsions levels, we cannot determine whether there is causal relationship between high blood glucose levels and convulsions in children with AGE. Large prospective studies are needed to address this question. Our study provides a solid evidence and rationale to conceive such studies.

The overall course of convulsions in our study was benign as it is evidenced by the negative CSF culture and mostly normal EEG in the cases who underwent these tests, as well as duration of and outcome of hospitalization, which is in agreement with previous studies^4,22,24^.

Potential mechanisms that might explain the occurrence of convulsions during an episode of AGE are not fully clear, and might include febrile seizures, neurological outcomes induced by *Shigella* infection via the mentioned above pathogen-host interactions^41–44^, neuro-virulence properties of enteric viruses^11,58,59^ and direct impact on CNS via viremia/antigenemia, for example in the case of rotavirus infection^58–60^, electrolyte imbalance (e.g., low serum sodium levels)^4,61^, or combination of these factors.

Our study has several strengths, including the multiyear data collection on both viral and bacterial AGE, large sample size, and having cases and controls from the same source population. Our study has limitations and our findings should be interpreted in view of these limitations. A limitation of our study is missing information on some of the study variables; this limitation was addressed by multiple imputation. We also did not have information on the exact timing of drawing blood sampling relative to hydration and onset of convulsions. Therefor if intravenous fluids were given prior to drawing blood samples, in such a scenario, the associations of electrolyte levels with convulsions might have been underestimated in our study. However, such a scenario is less likely, since usually blood biochemical tests are performed to support clinical decisions, including the administration of intravenous fluids. If misclassification of any of the study variables exists, it is non-differential and might to lead to underestimation of the measures of associations.

## Conclusions

Some children with AGE develop convulsions, mostly with a benign course, especially during the second year of life. Toddlers and those with shigellosis are at risk for convulsions during their diarrheal episode. High blood glucose in children with AGE and convulsion might be the result of neuroendocrine stress reaction. These findings contribute to further understanding the occurrence of convulsions in children with AGE.

## Consent to participate

Parents signed a written informed consent form.

## Consent for publication

Not applicable.

## Role of funders

We followed the World Health Organization generic protocol in the original study on acute gastroenteritis and rotavirus gastroenteritis study. Regarding the current study, the funders have no role in the study design, data collection, analysis and interpretation of data; in the writing of the report; and in the decision to submit the article for publication.

## Supplementary Information

Supplementary Information.

## Data Availability

Indiviual level data cannot be publicly available. Aggregative data might be provided upon a reasonable request to the corresponding author.

## References

[1] Collaborators GBDDD (2018). Estimates of the global, regional, and national morbidity, mortality, and aetiologies of diarrhoea in 195 countries: a systematic analysis for the Global Burden of Disease Study 2016. Lancet. Infect. Dis.

[2] Elliott EJ (2007). Acute gastroenteritis in children. Bmj.

[3] Zulfiqar AB (2015) Acute gastroenteritis in children. In: Kliegman R.M. SBF, St Geme J.W., Schor N.F (ed) Nelson Textbook of Pediatrics. Elsevier, Philadelphia, pp 1854–1874.

[4] Ma X, Luan S, Zhao Y, Lv X, Zhang R (2019). Clinical characteristics and follow-up of benign convulsions with mild gastroenteritis among children. Medicine.

[5] Kang B, Kwon YS (2014). Benign convulsion with mild gastroenteritis. Korean J. Pediatr..

[6] Dura-Trave T, Yoldi-Petri ME, Gallinas-Victoriano F, Molins-Castiella T (2011). Infantile convulsions with mild gastroenteritis: a retrospective study of 25 patients. Eur. J. Neurol..

[7] Ghorashi Z, Nezami N, Soltani-Ahari H, Ghorashi S (2010). Convulsion following gastroenteritis in children without severe electrolyte imbalance. Turk. J. Pediatr..

[8] Hiranrattana A, Mekmullica J, Chatsuwan T, Pancharoen C, Thisyakorn U (2005). Childhood shigellosis at King Chulalongkorn Memorial Hospital, Bangkok, Thailand: a 5-year review (1996–2000). Southeast Asian J Trop Med Public Health.

[9] Sethi SK, Khuffash FA, al-Nakib W (1989). Microbial etiology of acute gastroenteritis in hospitalized children in Kuwait. Pediatr. Infect. Dis. J..

[10] Solomon NH, Lavie S, Tenney BL, Blaser MJ (1982). Campylobacter enteritis presenting with convulsions. Clin. Pediatr..

[11] Chen SY, Tsai CN, Lai MW, Chen CY, Lin KL, Lin TY, Chiu CH (2009). Norovirus infection as a cause of diarrhea-associated benign infantile seizures. Clin. Infect. Dis..

[12] Verrotti A, Tocco AM, Coppola GG, Altobelli E, Chiarelli F (2009). Afebrile benign convulsions with mild gastroenteritis: a new entity?. Acta Neurol. Scand..

[13] Verrotti A, Moavero R, Vigevano F, Cantonetti L, Guerra A, Spezia E, Tricarico A, Nanni G, Agostinelli S, Chiarelli F, Parisi P, Capovilla G, Beccaria F, Spalice A, Coppola G, Franzoni E, Gentile V, Casellato S, Veggiotti P, Malgesini S, Crichiutti G, Balestri P, Grosso S, Zamponi N, Incorpora G, Savasta S, Costa P, Pruna D, Cusmai R (2014). Long-term follow-up in children with benign convulsions associated with gastroenteritis. Eur. J. Paediatric Neurol..

[14] Castellazzi L, Principi N, Agostoni C, Esposito S (2016). Benign convulsions in children with mild gastroenteritis. Eur. J. Paediatric Neurol..

[15] WHO (2020) Rotavirus immunization coverage estimates by country https://apps.who.int/gho/data/node.main.ROTACn?lang=en. January 9, 2021

[16] Muhsen K, Cohen D (2017). Rotavirus vaccines in Israel: uptake and impact. Hum. Vaccin. Immunother..

[17] Muhsen K, Anis E, Rubinstein U, Kassem E, Goren S, Shulman LM, Ephros M, Cohen D (2018). Effectiveness of rotavirus pentavalent vaccine under a universal immunization programme in Israel, 2011–2015: a case-control study. Clin. Microbiol. Infect..

[18] Muhsen K, Haklai Z, Applbaum Y, Gordon ES, Shteiman A, Glatman-Freedman A, Leshem E (2020). Effects of rotavirus vaccine on all-cause acute gastroenteritis and rotavirus hospitalizations in Israel: a nationwide analysis. Vaccine.

[19] Muhsen K, Kassem E, Rubenstein U, Goren S, Ephros M, Cohen D, Shulman LM (2016). Incidence of rotavirus gastroenteritis hospitalizations and genotypes, before and five years after introducing universal immunization in Israel. Vaccine.

[20] Muhsen K, Kassem E, Rubenstein U, Goren S, Ephros M, Shulman LM, Cohen D (2019). No evidence of an increase in the incidence of norovirus gastroenteritis hospitalizations in young children after the introduction of universal rotavirus immunization in Israel. Hum. Vaccin. Immunother..

[21] Muhsen K, Rubenstein U, Kassem E, Goren S, Schachter Y, Kremer A, Shulman LM, Ephros M, Cohen D (2015). A significant and consistent reduction in rotavirus gastroenteritis hospitalization of children under 5 years of age, following the introduction of universal rotavirus immunization in Israel. Hum. Vaccin. Immunother..

[22] Higuchi Y, Kubo T, Mitsuhashi T, Nakamura N, Yokota I, Komiyama O, Kamimaki I, Yamamoto S, Uchida Y, Watanabe K, Yamashita H, Tanaka S, Iguchi K, Ichimi R, Miyagawa S, Takayanagi T, Koga H, Shukuya A, Saito A, Horibe K (2017). Clinical epidemiology and treatment of febrile and afebrile convulsions with mild gastroenteritis: a multicenter study. Pediatr. Neurol..

[23] Ueda H, Tajiri H, Kimura S, Etani Y, Hosoi G, Maruyama T, Noma H, Kusumoto Y, Takano T, Baba Y, Nagai T (2015). Clinical characteristics of seizures associated with viral gastroenteritis in children. Epilepsy Res..

[24] Kang B, Kim DH, Hong YJ, Son BK, Kim DW, Kwon YS (2013). Comparison between febrile and afebrile seizures associated with mild rotavirus gastroenteritis. Seizure.

[25] Sara L, Simona M, Monica B, Francesca G, Stefano P (2016). Convulsions with gastroenteritis: reflections on some cases and tentative diagnostic score. Curr. Pediatr. Rev..

[26] Narchi H (2004). Benign afebrile cluster convulsions with gastroenteritis: an observational study. BMC Pediatr..

[27] Fasheh Youssef W, Pino Ramirez R, Campistol Plana J, Pineda Marfa M (2011). Benign afebrile convulsions in the course of mild acute gastroenteritis: a study of 28 patients and a literature review. Pediatr. Emerg. Care.

[28] Chen H, Zha J, Zhong JM, Chen Y, Yu XY, Xie JH (2019). Clinical features of campylobacter-associated benign convulsions with mild gastroenteritis compared with rotavirus convulsions. Seizure.

[29] Kim BR, Choi GE, Kim YO, Kim MJ, Song ES, Woo YJ (2018). Incidence and characteristics of norovirus-associated benign convulsions with mild gastroenteritis, in comparison with rotavirus ones. Brain Dev..

[30] Muhsen K, Shulman L, Rubinstein U, Kasem E, Kremer A, Goren S, Zilberstein I, Chodick G, Ephros M, Cohen D, TAU-HCLV Rota Study group (2009) Incidence, characteristics, and economic burden of rotavirus gastroenteritis associated with hospitalization of Israeli children <5 years of age, 2007–2008. J. Infect. Dis. 200 Suppl 1:S254–263. doi:10.1086/605425 10.1086/605425 19817606

[31] WHO (2002) Generic protocols for (i) hospital-based surveillance to estimate the burden of rotavirus gastroenteritis in children and (ii) a community-based survey on utilization of health care services for gastroenteritis in children.

[32] Israel Central Bureau of Statistics (2013) Characterization and classification of geographic units by the socio-economic level of the population 2008. State of Israel, Israel Central Bureau of Statistics, Jerusalem

[33] Ruuska T, Vesikari T (1990). Rotavirus Disease in Finnish Children: use of numerical scores for clinical severity of diarrheal episodes. Scand. J. Infect. Dis..

[34] Rubin DB (1996). Multiple imputation after 18+ years. J. Am. Stat. Assoc..

[35] Sterne JA, White IR, Carlin JB, Spratt M, Royston P, Kenward MG, Wood AM, Carpenter JR (2009). Multiple imputation for missing data in epidemiological and clinical research: potential and pitfalls. BMJ.

[36] Kavaliotis J, Karyda S, Konstantoula T, Kansouzidou A, Tsagaropoulou H (2000). Shigellosis of childhood in northern Greece: epidemiological, clinical and laboratory data of hospitalized patients during the period 1971–96. Scand. J. Infect. Dis..

[37] Shamsizadeh A, Nikfar R, Bavarsadian E (2012). Neurological manifestations of shigellosis in children in southwestern Iran. Pediatrics Int..

[38] Zvulunov A, Lerman M, Ashkenazi S, Weitz R, Nitzan M, Dinari G (1990). The prognosis of convulsions during childhood shigellosis. Eur. J. Pediatr..

[39] Ashkenazi S, Dinari G, Zevulunov A, Nitzan M (1987) Convulsions in childhood shigellosis. Clinical and laboratory features in 153 children. Am J Dis Child 141 (2):208–210. doi:10.1001/archpedi.1987.04460020098036 10.1001/archpedi.1987.04460020098036 3544808

[40] Balter-Seri J, Yuhas Y, Weizman A, Nofech-Mozes Y, Kaminsky E, Ashkenazi S (1999). Role of nitric oxide in the enhancement of pentylenetetrazole-induced seizures caused by Shigella dysenteriae. Infect Immun.

[41] Yuhas Y, Weizman A, Ashkenazi S (2003). Bidirectional concentration-dependent effects of tumor necrosis factor alpha in Shigella dysenteriae-related seizures. Infect. Immun..

[42] Yuhas Y, Shulman L, Weizman A, Kaminsky E, Vanichkin A, Ashkenazi S (1999). Involvement of tumor necrosis factor alpha and interleukin-1beta in enhancement of pentylenetetrazole-induced seizures caused by Shigella dysenteriae. Infect Immun..

[43] Yuhas Y, Weizman A, Dinari G, Ashkenazi S (1995). An animal-model for the study of neurotoxicity of bacterial products and application of the model to demonstrate that Shiga Toxin and lipopolysaccharide cooperate in inducing neurologic disorders. J. Infect. Dis..

[44] Yuhas Y, Nofech-Mozes Y, Weizman A, Ashkenazi S (2002). Enhancement of pentylenetetrazole-induced seizures by Shigella dysenteriae in LPS-resistant C3H/HeJ mice: role of the host response. Med Microbiol Immunol.

[45] Cohen D, Bassal R, Goren S, Rouach T, Taran D, Schemberg B, Peled N, Keness Y, Ken-Dror S, Vasilev V, Nissan I, Agmon V, Shohat T (2014). Recent trends in the epidemiology of shigellosis in Israel. Epidemiol. Infect..

[46] Svab D, Balint B, Vasarhelyi B, Maroti G, Toth I (2017). Comparative genomic and phylogenetic analysis of a shiga toxin producing Shigella sonnei (STSS) Strain. Front Cell Infect Microbiol.

[47] Nyholm O, Lienemann T, Halkilahti J, Mero S, Rimhanen-Finne R, Lehtinen V, Salmenlinna S, Siitonen A (2015). Characterization of Shigella sonnei isolate carrying shiga toxin 2-producing gene. Emerg Infect Dis.

[48] Lamba K, Nelson JA, Kimura AC, Poe A, Collins J, Kao AS, Cruz L, Inami G, Vaishampayan J, Garza A, Chaturvedi V, Vugia DJ (2016). Shiga toxin 1-producing shigella sonnei infections, California, United States, 2014–2015. Emerg Infect Dis.

[49] Sakai Y, Kira R, Torisu H, Yasumoto S, Saito M, Kusuhara K, Hara T (2006). Benign convulsion with mild gastroenteritis and benign familial infantile seizure. Epilepsy Res..

[50] Khan WA, Dhar U, Salam MA, Griffiths JK, Rand W, Bennish ML (1999). Central nervous system manifestations of childhood shigellosis: prevalence, risk factors, and outcome. Pediatrics.

[51] Gupta P, Natarajan G, Agarwal KN (1997). Transient hyperglycemia in acute childhood illnesses: to attend or ignore?. Indian J. Pediatr..

[52] Ronan A, Azad AK, Rahman O, Phillips RE, Bennish ML (1997). Hyperglycemia during childhood diarrhea. J. Pediatr..

[53] Seth A, Aneja S (1995). Hyperglycemia in malnourished children with dehydrating gastroenteritis. Indian J. Pediatr..

[54] Spirer Z, Bogair N (1970). Blood-sugars in infants with diarrhoea. Lancet.

[55] Ghobadifar MA, Honar N, Jooya P, Hassani F (2016). Blood glucose level after febrile convulsion. Korean J. Pediatr..

[56] Valerio G, Franzese A, Carlin E, Pecile P, Perini R, Tenore A (2001). High prevalence of stress hyperglycaemia in children with febrile seizures and traumatic injuries. Acta Paediatr..

[57] Costea RM, Maniu I, Dobrota L, Neamtu B (2020) Stress hyperglycemia as predictive factor of recurrence in children with febrile seizures. Brain Sci. 10 (3). doi:10.3390/brainsci10030131. 10.3390/brainsci10030131 PMC7139396 32120784

[58] Fischer TK, Ashley D, Kerin T, Reynolds-Hedmann E, Gentsch J, Widdowson MA, Westerman L, Puhr N, Turcios RM, Glass RI (2005). Rotavirus antigenemia in patients with acute gastroenteritis. J. Infect. Dis..

[59] Kehle J, Metzger-Boddien C, Tewald F, Wald M, Schüürmann J, Enders G (2003). First case of confirmed rotavirus meningoencephalitis in Germany. Pediatr. Infect. Dis. J..

[60] Chiappini E, Galli L, Martino Md (2006). Viremia and clinical manifestations in children with rotavirus infection. J. Infect. Dis..

[61] Motoyama M, Ichiyama T, Matsushige T, Kajimoto M, Shiraishi M, Furukawa S (2009). Clinical characteristics of benign convulsions with rotavirus gastroenteritis. J Child Neurol.

