# Supplementary material for: Convulsions in children hospitalized for acute gastroenteritis
Source: Sci Rep. 2021 Aug 5;11:15874. doi: 10.1038/s41598-021-95202-4 (PMC8342430; doi:10.1038/s41598-021-95202-4)
Supplement: Supplementary file 1 — Supplementary Information. [file 41598_2021_95202_MOESM1_ESM.pdf]

Convulsions in children hospitalized for acute gastroenteritis

Moti Iflah [1#], Eias Kassem[2#], Uri Rubinstein[3], Sophy Goren[4], Moshe Ephros[5,6], Dani Cohen[4] and Khitam Muhsen[4 *]

[1] School of Medicine, Sackler Faculty of Medicine, Tel Aviv University, Tel Aviv, Israel

[2] Department of Pediatrics, Hillel Yaffe Medical Center, Hadera, Israel

[3] Department of Pediatrics, Laniado Medical Center, Netanya, Israel

[4] Department of Epidemiology and Preventive Medicine, School of Public Health, Sackler Faculty of Medicine, Tel Aviv University, Tel Aviv, Israel

[5] Department of Pediatrics, Carmel Medical Center, Haifa, Israel

[6] Faculty of Medicine, Technion-Israel Institute of Technology, Haifa, Israel

[#] Contributed equally as first authors

* Correspondence: Khitam Muhsen, PhD, Department of Epidemiology and Preventive Medicine, School of Public Health, Sackler Faculty of Medicine, Tel Aviv University, Ramat Aviv, Tel Aviv, Israel, 6139001; Telephone +972-3-6405945, Fax 972-6409868. E-mail: kmuhsen@tauex.tau.ac.il

Supplementary material

Supplementary Table 1: Number and percent of missing information on selected variables.

Variable	*Total*	*Number valid*	*Number with missing*	*% with missing*
Gestational age at birth	161	103	58	36.0
Birth weight	161	95	66	41.0
Leukocytes	161	151	10	6.2
Hemoglobin	161	151	10	6.2
Platelets	161	151	10	6.2
Glucose	161	148	13	8.1
Potassium	161	144	17	10.6
Sodium	161	147	14	8.7
C-reactive protein	161	147	14	8.7

Supplementary Table 2: Socio-demographic characteristics of children hospitalized for acute gastroenteritis with convulsions (cases) and without convulsions (controls).

	Cases (n=47)	*Controls (n=114)*	*P value*
Age, months			0.542
0-11	7 (15%)	21 (18%)	
12-23	28 (60%)	57 (50%)	
24-59	12 (25%)	36 (32%)	
**Sex**			0.902
Males	23 (49%)	57 (50%)	
Females	24 (51%)	57 (50%)	
Population groups			0.732
Arabs	22 (46.8%)	50 (44%)	
Jews	25 (53.2%)	64 (56%)	
SES of place of residence [a]			0.373
1-3 (Low)	12/39 (31%)	42/101 (42%)	
4-5 (Intermediate)	20/39 (51%)	39/101 (39%)	
6-10 (High)	7/39 (18%)	20/101 (19%)	

P value was obtained by the chi square test. [a] SES: socioeconomic status. Information on SES of place of residence was missing for 8 (17%) cases and 13 (11%) controls.

Supplementary Table 3: Adjusted associations of clinical characteristics with convulsions in children hospitalized for acute gastroenteritis-Complete case analysis.

Variable	*Adjusted OR (95% CI)*	*P value*
Gestational age at birth, weeks	0.75 (0.54-1.04)	0.087
C-reactive protein, mg/L	0.99 (0.97-1.00)	0.091
Body temperature, °C	3.12 (1.66-5.87)	<0.001
Hyponatremia (Sodium <135 mEq/L)	3.87 (0.83-18.04)	0.085
High plasma glucose(>120 mg/dL)	7.93 (1.36-45.77)	0.020
Severe gastroenteritis (Vesikari score ≥11)	0.19 (0.06-0.63)	0.007

CI: confidence intervals; OR: odds ratio. Results were obtained by multiple logistic regression model.

Supplementary Table 4: Adjusted associations of clinical characteristics with convulsions in children hospitalized for acute gastroenteritis [analysis limited to children with fever (>38 °C), 45 cases and 80 controls)

Variable	*Adjusted OR (95% CI)*	*P value*
Gestational age at birth, weeks	0.83 (0.63-1.09)	0.171
C-reactive protein, mg/L	0.99 (0.97-1.00)	0.078
Body temperature, °C	2.40 (1.20-4.78)	0.013
Hyponatremia (Sodium <135 mEq/L)	4.42 (1.07-18.20)	0.040
High plasma glucose(>120 mg/dL)	5.76 (1.05-31.69)	0.044
Severe gastroenteritis (Vesikari score ≥11)	0.08 (0.03-0.22)	<0.001

CI: confidence intervals; OR: odds ratio. Results were obtained by multiple logistic regression model.
